# Cocaine Enhances HIV-1 Transcription in Macrophages by Inducing p38 MAPK Phosphorylation

**DOI:** 10.3389/fmicb.2016.00823

**Published:** 2016-06-09

**Authors:** Chelsie Swepson, Alok Ranjan, Muthukumar Balasubramaniam, Jui Pandhare, Chandravanu Dash

**Affiliations:** ^1^Center for AIDS Health Disparities Research, Meharry Medical College, NashvilleTN, USA; ^2^Department of Biochemistry and Cancer Biology, Meharry Medical College, NashvilleTN, USA; ^3^School of Graduate Studies and Research, Meharry Medical College, NashvilleTN, USA; ^4^Department of Microbiology and Immunology, Meharry Medical College, NashvilleTN, USA

**Keywords:** cocaine, HIV, p38 MAPK, MSK1, macrophages

## Abstract

Cocaine is a commonly used illicit drug among HIV-1 infected individuals and is known to increase HIV-1 replication in permissive cells including PBMCs, CD4^+^ T cells, and macrophages. Cocaine’s potentiating effects on HIV-1 replication in macrophages- the primary targets of the virus in the central nervous system, has been suggested to play an important role in HIV-1 neuro-pathogenesis. However, the mechanism by which cocaine enhances HIV-1 replication in macrophages remain poorly understood. Here, we report the identification of cocaine-induced signaling events that lead to enhanced HIV-1 transcription in macrophages. Treatment of physiologically relevant concentrations of cocaine enhanced HIV-1 transcription in a dose-dependent manner in infected THP-1 monocyte-derived macrophages (THP-1macs) and primary monocyte-derived macrophages (MDMs). Toward decoding the underlying mechanism, results presented in this report demonstrate that cocaine induces the phosphorylation of p38 mitogen activated protein kinase (p38 MAPK), a known activator of HIV-1 transcription. We also present data suggesting that the p38 MAPK-driven HIV-1 transcription is dependent on the induction of mitogen- and stress-activated protein kinase 1 (MSK1). Consequently, MSK1 mediates the phosphorylation of serine 10 residue of histone 3 (H3 Ser10), which is known to activate transcription of genes including that of HIV-1 in macrophages. Importantly, our results show that inhibition of p38 MAPK/MSK1 signaling by specific pharmacological inhibitors abrogated the positive effect of cocaine on HIV-1 transcription. These results validate the functional link between cocaine and p38 MAPK/MSK1 pathways. Together, our results demonstrate for the first time that the p38 MAPK/MSK1 signaling pathway plays a critical role in the cocaine-induced potentiating effects on HIV-1 infection, thus providing new insights into the interplay between cocaine abuse and HIV-1 neuro-pathogenesis.

## Introduction

The Central Nervous System (CNS) is a major target of Human Immunodeficiency Virus type 1 (HIV-1; McArthur et al., 2010). HIV-1 enters the CNS early in infection and leads to progressive neuronal dysfunction and degeneration (Kaul et al., 2001; Nath, 2002; Rao et al., 2014) that collectively constitute HIV-associated neurological disorders (HAND; Woods et al., 2009; Patel et al., 2013; Sanmarti et al., 2014). HAND affects 20–30% patients in the late stages of AIDS and is believed to be the most common cause of dementia worldwide among people aged 40 and below (Vivithanaporn et al., 2010). While antiretroviral therapy (ART) has dramatically reduced HIV-1 associated dementia (Joska et al., 2010), the milder forms of HAND continues to be a major health concern for HIV-1 patients (Joska et al., 2010). Cocaine, a commonly abused drug among HIV-1 positive individuals, has been associated with worsening of HAND pathogenesis (Pakesch et al., 1992; Larrat and Zierler, 1993; Goodwin et al., 1996; Nath et al., 2001; Ferris et al., 2008; Norman et al., 2009). Several mechanisms have been proposed for the potentiating effects of cocaine on HAND including enhanced neuro-invasion of HIV-1, increased viral replication in the CNS, and elevated toxic effects of HIV-1 viral proteins (Pakesch et al., 1992; Larrat and Zierler, 1993; Goodwin et al., 1996; Nath et al., 2001; Ferris et al., 2008; Norman et al., 2009). Though neurons are generally refractory to HIV-1 (van de Bovenkamp et al., 2002), resident macrophages/microglia support HIV-1 infection and serve as a reservoir for the virus in the CNS (Koppensteiner et al., 2012). Ongoing viral replication in these cells are hypothesized to drive HIV-1 neuro-pathogenesis by releasing viral and cellular factors that induce neuroinflammation and neurotoxicity (Gonzalez-Scarano and Martin-Garcia, 2005; Rao et al., 2014). However, the exact mechanism(s) by which cocaine potentiates HIV-1 replication in macrophages is poorly understood.

Cocaine enhances HIV-1 replication in various permissive cell types including peripheral blood mononuclear cells (PBMCs; Peterson et al., 1991, 1992; Bagasra and Pomerantz, 1993), CD4^+^ T cells (Mantri et al., 2012; Kim et al., 2013; Addai et al., 2015), dendritic cells (Napuri et al., 2013), and macrophages (Dhillon et al., 2007). These studies suggest that to increase HIV-1 replication cocaine targets both entry and post-entry steps of infection. It has been reported that, cocaine promotes the entry of HIV-1 into target cells by inhibiting anti-viral chemokines and upregulating the co-receptors (CXCR4 and/or CCR5) that mediate viral entry and fusion (Nair et al., 2000, 2001; Roth et al., 2005; Reynolds et al., 2009). Studies also support that cocaine alters HIV-1 post entry steps including reverse transcription, integration, and viral mRNA transcription. Recently, we have demonstrated that in CD4^+^ T cells cocaine downregulates “miR-125b” (Mantri et al., 2012), an anti-HIV cellular miRNA that inhibits HIV-1 protein translation by binding to viral transcripts (Huang et al., 2007). Cocaine also downregulates another cellular miRNA called miR-155 (Napuri et al., 2013), which negatively regulates DC-SIGN that plays a critical role in HIV-1 infection of dendritic cells (Napuri et al., 2013). Cocaine has also been shown to enhance HIV-1 reverse transcription in quiescent CD4^+^ T cells (Kim et al., 2013). Along these lines, very recently, we reported that cocaine treatment increases HIV-1 proviral integration in CD4^+^ T cells (Addai et al., 2015). Further, proteomic analysis of cocaine-treated astrocytes revealed differential regulation of several host proteins that may potentially influence HIV-1 replication (Reynolds et al., 2006). Collectively, these studies highlight the potential mechanisms by which cocaine might modulate both entry and post entry steps of HIV-1 replication in PBMCs, CD4^+^ T cells, astrocytes, and dendritic cells. However, the cellular pathways and the steps of replication that are targeted by cocaine in macrophages remain poorly understood.

The goal of this study was to decipher the mechanism by which cocaine increases HIV-1 replication in macrophages. HIV-1 replication can be modulated by cellular signaling pathways known to stimulate HIV-1 LTR driven transcription (Herbein et al., 2010). In macrophages, activation of nuclear factor kappa B (NF-κB), mitogen activated protein kinases (MAPK), and Janus kinase/signal transducer and activator of transcription (JAK/STAT) have been reported to stimulate HIV-1 transcription (Herbein et al., 2010). Cocaine has also been reported to alter cellular gene expression by targeting MAPK, PI3K/Akt pathway, and mitogen- and stress-activated protein kinases 1 (MSK1; Yao et al., 2009; Yang et al., 2010; Mannangatti et al., 2015). In the present study, we demonstrate that cocaine enhances HIV-1 transcription in macrophages by targeting the p38 MAPK/MSK1 pathway. Using THP-1macs and primary MDMs, we show that cocaine induces the phosphorylation of p38 MAPK, which then activates the MSK1 pathway that subsequently promotes the phosphorylation of serine 10 residue of histone 3 (H3 Ser10). We also demonstrate that inhibition of p38 MAPK/MSK1 pathway abrogates cocaine’s positive effects on HIV-1 transcription. Together, our results strongly implicate the p38 MAPK/MSK1 signaling in cocaine-treated macrophages and provide mechanistic insights into the potentiating effects of this illicit drug on HIV-1 replication.

## Materials and Methods

### Reagents and Antibodies

Cocaine Hydrochloride was purchased from Sigma Chemicals (St. Louis, MO, USA). P38 MAPK inhibitor-SB203580, Anti-p38 and anti-phospho-p38 antibodies were purchased from Cell Signaling Technology (Danvers, MA, USA). The MSK1 inhibitor SB747651A was purchased from R&D Systems (Minneapolis, MN, USA). Anti-phospho p38-Alexa 647 conjugate was purchased from Beckman Coulter (Brea, CA, USA). Anti-MSK1, anti Phospho-MSK1, anti-Histone H_3,_ and anti-phospho-Ser 10 Histone H_3_ were obtained from Millipore (Billerica, MA, USA) and anti-actin antibody was obtained from Sigma (St. Louis, MO, USA). The plasmids pLVSV-G and pNL4-3.Luc.R^-^E^-^ were gifts from Dr. Vineet KewalRamani (NCI/NIH). The following reagent were obtained through the NIH AIDS Research and Reference Reagent Program, Division of AIDS, NIAID, NIH: TZM-bl from Dr. John C. Kappes, Dr. Xiaoyun Wu and Tranzyme Inc. The monocytic THP-1 cell line was obtained from American Type Culture Collection (ATCC). Whole human blood was purchased from the New York Blood Center in accordance with Meharry Medical College IRB guidelines.

### Cell Culture, Isolation of PBMCs, Purification of Primary Monocytes, and Generation of Monocyte-Derived Macrophages (MDMs)

THP-1 cell line was maintained in complete RPMI (cRPMI) media containing RPMI supplemented with 10% heat-inactivated fetal bovine serum (FBS), 2 mM L-glutamine, and antibiotics (100 U/ml penicillin, 100 μg/ml streptomycin). THP-1 monocytes were differentiated into macrophages (THP-1macs) by culturing them with 10 ng/mL of phorbol 12-myristate 13-acetate (PMA) for 72 h.

For primary cell isolation, fresh human blood was diluted 1:2 with phosphate buffered saline (PBS), overlaid on 12.5 ml of Ficoll-Paque^TM^ Premium reagent (GE) in a 50 ml conical tube, and centrifuged at 750 × *g* for 20 min at 20°C. The interphase layer of human PBMCs was carefully transferred to a new 50 ml conical tube and PBS was added to make up to 50 ml. Subsequently, the PBMCs were washed several times with PBS by centrifugation to remove unwanted cell types. The resulting cell pellet was resuspended in PBS, and the cell number and viability were determined by trypan blue exclusion. Monocytes were then isolated from the PBMCs by negative selection using the Monocyte Isolation kit II (Miltenyi Biotec) and following the manufacturer-recommended protocol. The isolated monocytes were cultured in RPMI 1640 supplemented with 20% heat-inactivated FBS, 2 mM L-glutamine, and antibiotics. These monocytes were then differentiated to macrophages (MDMs) by culturing them in 10 ng/ml M-CSF (10 ng/ml; Life Technologies, Carlsbad, CA, USA) for 5–7 days.

### Virus Production and Infection

VSV-G-pseudotyped HIV-1 encoding the firefly luciferase was generated by co-transfecting the HIV-1 molecular clone pNL4-3.Luc.R^-^E^-^ and pLVSV-G into 293T cells by using Polyfect (Qiagen) as per the manufacturer-recommended protocol. After 48–72 h, culture supernatant containing the virus particles was collected, centrifuged at low-speed and filtered through a 0.45 μM-pore-size membrane. Infectivity of the virions was measured by luciferase reporter assay using TZM-bl cells that harbor a firefly luciferase reporter gene under the control of HIV-1 promoter. THP-1macs and MDMs (5 × 10^4^ cells) were infected with VSV-G-pseudotyped HIV-1 by spinoculation. Six hours post-infection, cells were washed with PBS, treated with varying concentrations of cocaine, and incubated at 37°C. After 48–72 h, cells were washed, lysed, and luciferase activity was measured using a luminescence microplate reader (BioTek).

HIV-1 BAL virions were obtained from the NIH AIDS Reagent Program, Division of AIDS, NIAID, NIH. THP-1macs were infected with HIV-1 BAL virions (MOI ∼ 1.0) by spinoculation in the presence of polybrene (Sigma) and were cultured (4 × 10^5^ cells/ml) for 3 days in the presence or absence of cocaine. Productive infection was measured by detecting intracellular HIV-1 p24 protein using western blot. Activation of p38 MAPK/MSK1 pathway in infected cells was measured by western blot using cellular lysates of infected cells treated with or without cocaine.

### Western Blotting

Cell lysates were prepared using standard protocols and the total protein concentrations were determined by BCA protein assay. Equal amounts of total protein from the cell lysates were resolved by SDS-PAGE and then transferred to nitrocellulose membranes by using a semi-dry blotter (Bio-Rad, Hercules, CA, USA). The membranes were incubated in 5% (w/v) non-fat milk in TBST (10 mM Tris. pH 8.0, 150 mM NaCl, and 0.1% Tween 20). Membranes were then probed with primary antibodies recognizing antigens of interest, followed by incubation with appropriate secondary antibodies (1:2000 dilution) conjugated to horseradish peroxidase. Immunoblots were washed in TBST (pH 8.0; Sigma) and developed using the enhanced chemiluminescence system (Pierce ECL, Thermo Scientific).

### Flow Cytometry Analysis

For intracellular staining of phosphorylated p38-MAPK and MSK1, THP-1macs and MDMs were harvested by scraping with 10% (w/v) StemPro Accutase cell detachment solution (Life Technologies, Carlsbad, CA, USA) and collected by centrifugation. The cells were then washed with PBS and fixed in 4% (w/v) paraformaldehyde at 4°C for 15 min. Cells were then incubated on ice for 1 min and washed with PBS. Following fixation, cells were permeabilized in 90% (w/v) methanol for 30 min on ice. Cells were washed and re-suspended in PBS containing 0.5% BSA. Upon washing, the cells were stained for phospho-p38 and phospho-MSK1 using Alexa Fluor 647 rabbit anti-phospho-p38 and anti-phospho-MSK1 antibodies (BD Biosciences) according to the manufacturer’s instructions. Flow cytometry was performed after a final wash (PBS containing 0.5% BSA) using a BD FACSCalibur^TM^ flow cytometer (BD Biosciences) and data were collected and analyzed using Flow Jo (Tristar) software.

### Cytotoxicity Assay

Cells were treated with cocaine for 24 h and cytotoxicity was measured by using Vybrant MTT Cell Proliferation Assay Kit as per the manufacturers’ instructions (Thermo Fisher, USA).

### Statistical Analysis

Data are analyzed by one-way analysis of variance (ANOVA) and presented as mean ± SD of three independent experiments. Significance of differences between control and cocaine-treated samples was determined by Turkey *post hoc* test. Values of *p* < 0.05 were considered to be statistically significant.

## Results

### Cocaine Enhances HIV-1 Transcription in Macrophages

**FIGURE 1:**
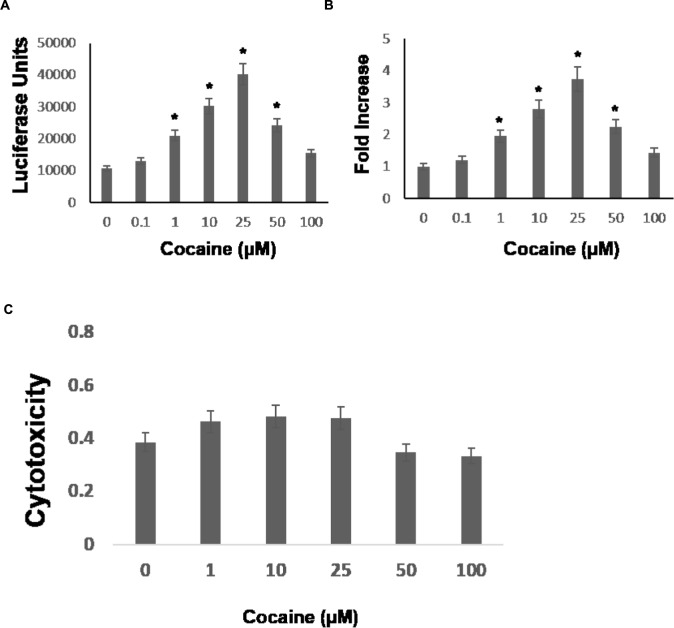
**Cocaine increases HIV-1 transcription in THP-1macs.** THP-1macs were inoculated with pseudotyped HIV-1 encoding an LTR-driven luciferase reporter and then were cultured in the absence or presence of cocaine at concentrations ranging from 0.1 to 100 μM to cover the wide range of plasma concentrations reported in cocaine users. **(A)** Viral replication was monitored 48–72 hpi by measuring luciferase activity in the cell lysates. **(B)** Fold change in viral replication was determined by comparing the luciferase activity of cocaine-treated cells to that of the untreated cells. **(C)** THP-1macs were cultured in the absence or presence of cocaine at concentrations ranging from 0.1 to 100 μM. Cytotoxicity was measured by optical density at 570 nm using MTT based assay. The results are expressed as mean ± SE for three independent experiments. ^∗^*p* < 0.05 is for the comparison of cocaine treated cells vs. untreated cells.

Cocaine is known to enhance HIV-1 replication in permissive cell types including macrophages. Macrophages express the CCR5 co-receptor (Grivel et al., 2011) and cocaine has been shown to upregulate CCR5 expression (Nair et al., 2000). Because HIV-1 entry into macrophages is mediated by the cellular receptor CD4 and the co-receptors-CCR5 (Grivel et al., 2011), it has been suggested that cocaine’s potentiating effect on HIV-1 in macrophages may depend on increased viral entry. We have previously demonstrated that cocaine targets post-entry steps of HIV-1, which causes increased viral replication in CD4^+^ T cells [30, 31, 32]. To test whether cocaine modulates post entry steps of HIV-1 infection in macrophages, we performed single-cycle replication assays using a luciferase encoding HIV-1 reporter virus. The THP-1macs were infected with HIV-1 virions by spinoculation and then cultured in the presence of cocaine at concentrations ranging from 0.1 to 100 μM. These concentrations cover the wide range of plasma levels of cocaine reported in drug users (Van Dyke et al., 1976; Mittleman and Wetli, 1984; Peretti et al., 1990; Karch et al., 1998; Blaho et al., 2000; Heard et al., 2008). The viral replication levels were evaluated by lysing the cells 48–72 h post infection (hpi) and measuring luciferase activity in the cell lysates. As shown in **Figure 1A**, cocaine treatment increased HIV-1 promoter driven luciferase activity in THP-1macs compared to untreated infected cells. Importantly, increasing concentrations of cocaine caused a dose-dependent increase in luciferase activity (**Figure 1A**), with 25 μM cocaine producing the maximum activity corresponding to ∼threefold increase in viral replication compared to untreated cells (**Figure 1B**). Cytotoxicity measurements revealed that treatment of cocaine at concentrations of 1 μM through 100 μM exhibited minimal toxicity to THP-1macs (**Figure 1C**).

### Cocaine Activates p38 MAPK in Macrophages

**FIGURE 2:**
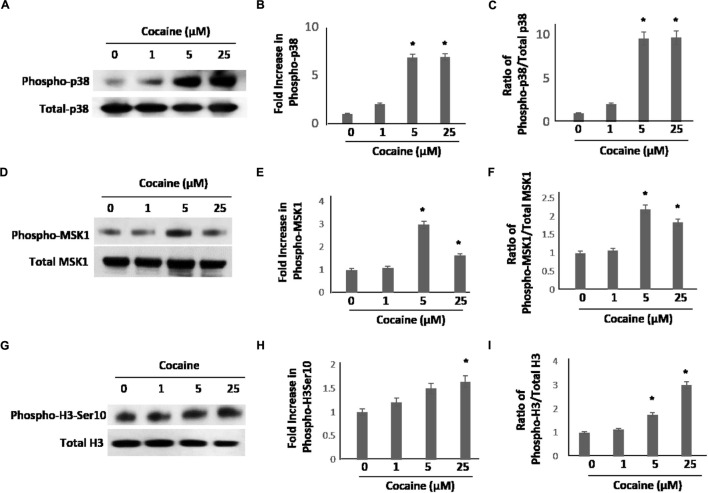
**Cocaine induces p38 MAPK/MSK1/H3-Ser10 signaling cascade in uninfected THP-1macs.** THP-1macs were cultured in the presence of increasing concentrations of cocaine overnight. Cells were harvested and levels of p38 MAPK, MSK1, and H3 Ser10 were assessed. **(A)** Levels of total p38 and phospho-p38 by western blot analysis. Densitometry analysis of fold change in phospho-p38 levels in cocaine-treated cells relative to untreated cells **(B)**, and fold change in phospho-p38 levels relative to total p38 for respective cocaine concentrations **(C)**. **(D)** Levels of total MSK1 and phospho-MSK1 by western blot analysis. Densitometry analysis of fold change in phospho-MSK1 levels in cocaine-treated cells relative to untreated cells **(E)**, and fold change in phospho-MSK1 levels relative to total MSK1 for respective cocaine concentrations **(F)**. **(G)** Levels of total H3 and phospho-H3 Ser10 by western blot analysis. Densitometry analysis of fold change in phospho-H3 Ser10 levels in cocaine-treated cells relative to the untreated cells **(H)**, and fold change in phospho-H3 Ser10 levels relative to total H3 for respective cocaine concentrations **(I)**. The results are expressed as mean ± SE for three independent experiments. ^∗^*p* < 0.05 is for the comparison of cocaine treated cells vs. untreated cells.

As cocaine treatment potentiated the HIV-1 LTR promoter-driven transcription, we sought to understand the underlying mechanistic details. We focused on the p38 MAPK pathway based on two lines of published data showing that: (1) cocaine activates p38 MAPK in neurons and astrocytes (Yao et al., 2009; Yang et al., 2010), and (2) p38 MAPK activates HIV-1 LTR-driven transcription in monocytic cell lines and macrophages (Muthumani et al., 2004; Horie et al., 2007; Kadoki et al., 2010; Sahu et al., 2015). To test this, first we investigated whether cocaine activates p38 MAPK in macrophages. We treated THP-1macs with 1, 5, and 25 μM cocaine, concentrations that stimulated HIV-1 transcription in these cells (**Figure 1**). After cocaine treatment, cells were lysed and expression levels of total p38 and phosphorylated p38 (phospho-p38) were examined by immunoblot. Data from these experiments show that cocaine treatment significantly enhanced phospho-p38 levels relative to the untreated cells (**Figure 2A**). Densitometry analysis revealed a ∼sixfold to sevenfold increase in phospho-p38 levels in cells treated with 5 and 25 μM cocaine compared to untreated cells (**Figure 2B**). Determination of the ratio between the phospho-p38 level to the total p38 MAPK level for respective cocaine concentrations revealed a ∼eightfold to 10-fold increase in phosphorylation of p38 MAPK in cells treated with 5 and 25 μM cocaine (**Figure 2C**). Importantly, the maximum increase in phospho-p38 levels at 25 μM cocaine correlated with the greatest increase in HIV-1 transcription induced by 25 μM cocaine (**Figure 1**). These data strongly indicate that cocaine targets and activates the p38 MAPK in macrophages.

### Cocaine-Mediated Phosphorylation of p38 MAPK Leads to the Activation of MSK1

Having determined that cocaine induces phosphorylation of p38 MAPK (**Figure 2A**), we then sought to identify the downstream effectors of p38-MAPK in cocaine-treated macrophages. We chose to evaluate the MSK1, because it is a known target of p38 MAPK (Vermeulen et al., 2009) and is also activated by cocaine (Brami-Cherrier et al., 2005). To test this, THP-1macs were treated with 1, 5, and 25 μM cocaine and the lysates of these cells were analyzed for the activation of MSK1 by measuring phosphorylation of MSK1 by western blot. As shown in **Figure 2D**, cells exposed to cocaine exhibited increased phospho-MSK1 levels compared to the untreated cells. Densitometry analysis showed that the increase in MSK1 phosphorylation was optimal (∼twofold to threefold) in cells treated with 5 μM cocaine (**Figure 2E**). The p38 MAPK activity, expressed as the ratio of phosphorylated to total p38 MAPK levels, rose to the maximum (∼ twofold) in cells treated with 5 or 25 μM cocaine (**Figure 2F**). These results indicate that the p38 MAPK/MSK1 pathway may be an important determinant of cocaine-mediated signaling in THP-1macs.

### Cocaine-Induced Activation of p38 MAPK/MSK1 Pathway Stimulates Phosphorylation of Histone H3 at Ser10

Mitogen- and stress-activated protein kinases 1 is a key signaling molecule downstream of p38 MAPK and is known to induce phosphorylation of the serine 10 of histone H3 (H3 Ser10; McCoy et al., 2005, 2007). Studies have demonstrated that MSK1 is required for stress-induced phosphorylation of H3 Ser10 resulting in transcriptional activation of cellular genes (Keum et al., 2013). Interestingly, MSK1/H3 Ser10 has also been reported to drive viral transcription (Sahu et al., 2015). Therefore, we hypothesized that cocaine-mediated activation of MSK1 signaling could lead to phosphorylation and activation of H3 Ser10. To test this, we measured the phosphorylation of H3 Ser10 in cocaine-treated cell lysates of THP-1macs by western blot. Data in **Figure 2G** showed that cocaine treatment (18–24 h) increased the H3 phospho-Ser10 levels in a dose dependent manner. In densitometry analysis, cells treated with 25 μM cocaine exhibited the highest levels of H3 phospho-Ser10 when compared to the untreated cells (**Figure 2H**). The ratio of H3 phospho-Ser10 to the total H3 Ser10 also indicated that 25 μM cocaine caused the greatest (threefold) increase in phosphorylation (**Figure 2I**). Taken together, these data suggest that the p38 MAPK/MSK1 pathway promotes phosphorylation and activation of H3 Ser10 in cocaine- treated THP-1macs.

### Inhibiting p38 MAPK/MSK1 Pathway Abrogates Cocaine-Mediated Effects on HIV-1 Transcription

**FIGURE 3:**
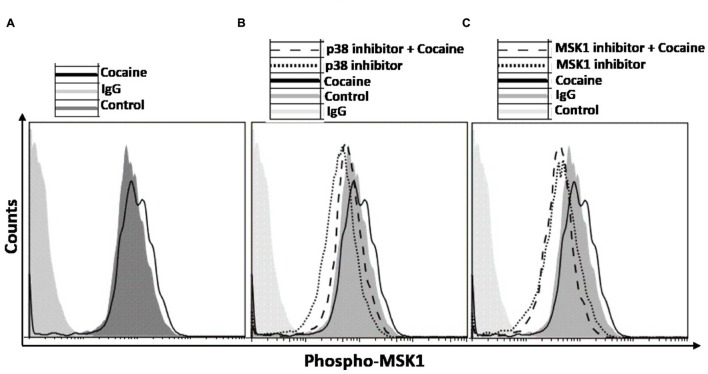
**Induction of p38 MAPK/MSK1 signaling cascade in uninfected THP-1macs by cocaine can be abrogated by pharmacological inhibitors of p38 MAPK and MSK1.** THP-1macs were pretreated with 10 μM p38 MAPK inhibitor (SB-203580) or 10 μM MSK1 inhibitor (SB-747651A) for 1 h prior to overnight treatment with cocaine. Cells were then harvested, stained with appropriate antibodies or isotype controls, and analyzed by flow cytometry. **(A)** Levels of phospho-MSK1 in cocaine-treated cells. Levels of phospho-MSK1 in cocaine- and p38 MAPK inhibitor-treated cells **(B)**, and in cocaine- and MSK1 inhibitor-treated cells **(C)**.

We probed the specificity of the induction of p38 MAPK/MSK1 pathway in cocaine treated THP1macs by using the pharmacological inhibitors specific for p38 MAPK (SB-203580; Davies et al., 2000) and MSK1 (SB-747651A; Naqvi et al., 2012). THP1macs were pretreated with either DMSO, or p38 MAPK inhibitor (10 μM) or MSK1 inhibitor (10 μM) prior to cocaine (25 μM) treatment (**Figure 3B**). Then, flow cytometry was used to analyze the phosphorylation levels of MSK1 and thus its activation status. As hypothesized, in the absence of p38 MAPK inhibitor, cocaine increased the phosphorylation levels of MSK1 in THP1macs (**Figure 3A**). In contrast, in THP-1macs pretreated with the p38 MAPK inhibitor, cocaine had only a minimal effect on the phosphorylation status of MSK1 (**Figure 3B**). Likewise, cocaine failed to induce MSK1 phosphorylation in cells treated with MSK1 inhibitor (**Figure 3C**). However, in the absence of the MSK1 inhibitor, cocaine induced MSK1 phosphorylation (**Figure 3C**). The results of these experiments provide further evidence that cocaine specifically targets the p38 MAPK/MSK1 pathway in THP-1macs.

### Cocaine-Induced Activation of p38 MAPK/MSK1 Signaling Leads to Enhanced Transcription of HIV-1 in Macrophages

**FIGURE 4:**
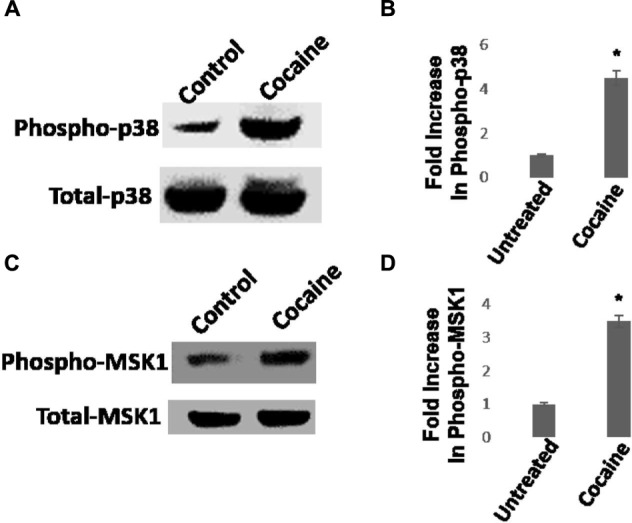
**Cocaine induces p38 MAPK/MSK1 signaling in HIV-1-infected THP-1macs.** **(A)** THP-1macs were inoculated with pseudotyped HIV-1 and then cultured in the absence or presence of 25 μM cocaine. Cells were harvested 48 hpi and cell lysates were analyzed by western blot. **(A)** Levels of total p38 MAPK and phospho-p38 MAPK. **(B)** Fold change in phospho-p38 MAPK levels in cocaine-treated cells relative to untreated cells. **(C)** Levels of total MSK1 and phospho-MSK1 levels. **(D)** Fold change in phospho-MSK1 levels in cocaine-treated cells relative to untreated cells. The results in **(B,D)** are expressed as mean ± SE for three independent experiments. ^∗^*p* < 0.05 is for the comparison of cocaine treated cells vs. untreated cells.

We then investigated whether cocaine activates the p38 MAPK/MSK1 pathway in HIV-1-infected THP-1macs. Cells were inoculated with VSV-G-pseudotyped HIV-1-Luc reporter virions and then cultured in the presence or absence of cocaine (25 μM). Productive HIV-1 infection in THP-1macs was confirmed by measuring luciferase activity in the cell lysates (data not shown). Data from western blot analysis showed that cocaine stimulated the phosphorylation of p38 MAPK (**Figures 4A,B**) and MSK1 (**Figures 4C,D**) in HIV-1-infected THP1macs.

**FIGURE 5:**
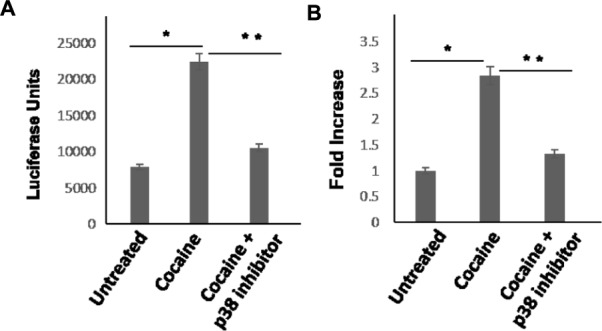
**Cocaine-induced p38 MAPK signaling enhances HIV-1 transcription in THP-1macs.** THP-1macs were inoculated with pseudotyped HIV-1 and then were cultured in the absence or presence of 25 μM cocaine with or without pretreatment with 10 μM p38 MAPK inhibitor (SB-203580). **(A)** Viral replication was monitored 48–72 hpi by measuring luciferase activity in the cell lysates. **(B)** Fold change in viral replication was determined by comparing the luciferase activity of cocaine-treated cells to that of the untreated cells. The results are expressed as mean ± SE for three independent experiments. ^∗^*p* < 0.05 is for the comparison of cocaine treated cells vs. untreated cells. ^∗∗^*p* < 0.05 is for the comparison of cocaine treated cells vs. cocaine and inhibitor treated cells.

Next, we tested whether activation of the p38 MAPK and MSK1 pathways is essential for the cocaine-induced stimulation of HIV-1 transcription in THP-1macs (**Figures 1A,B**). To test this possibility, THP-1macs were inoculated with VSV-G-pseudotyped HIV-1-Luc reporter virions. Then these cells were pretreated with p38 MAPK inhibitor or DMSO for 1 h, and then cultured in the presence or absence of cocaine (25 μM). Recapitulating the data shown in **Figure 1**, HIV-1 transcription, as evidenced from the luciferase activity, was significantly elevated in THP-1macs pretreated with DMSO and cultured in the presence of cocaine, compared to control cells (**Figures 5A,B**). In contrast, a significant decrease in luciferase activity was observed in THP-1macs pretreated with p38 MAPK inhibitor and cultured in the presence of cocaine. These results confirm the functional role of the p38 MAPK/MSK1 pathway in the cocaine-induced enhancement of HIV-1 transcription in THP-1macs.

**FIGURE 6:**
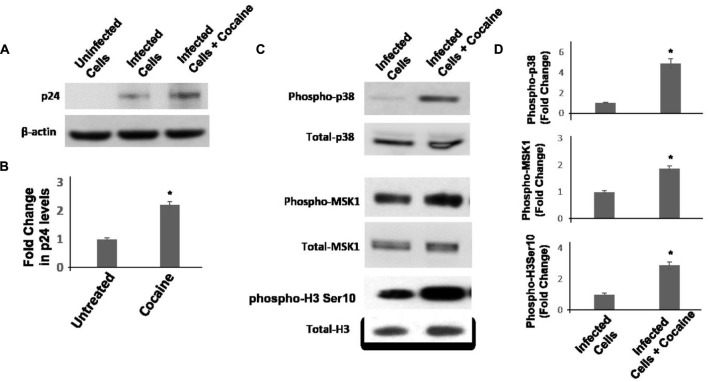
**Cocaine activates p38 MAPK/MSK1/H3Ser10 signaling in THP-1macs infected with R5 tropic HIV-1 BAL virions.** THP-1macs were inoculated with HIV-1 BAL virions and spinoculated for 2 h. These cells were then cultured with or without cocaine (25 μM). **(A)** Viral replication was monitored 72 hpi by measuring HIV-1 p24 protein in the cell lysates by immunoblot. **(B)** Fold change in p24 levels in cocaine-treated cells relative to untreated cells. **(C)** Levels of total p38 MAPK and phospho-p38 MAPK, total MSK1 and phosphor-MSK1, and total H3 Ser10, and phosphor-H3 Ser10 were measured in the lysates by immunoblot. **(D)** Fold change in respective phosphorylated protein levels in cocaine-treated cells relative to untreated cells. The results are expressed as mean ± SE for three independent experiments. ^∗^*p* < 0.05 is for the comparison of cocaine treated cells vs. untreated cells.

Result in **Figures 4** and **5** support the hypothesis that cocaine enhances HIV-1 transcription in macrophages by activating the p38 MAPK/MSK1 signaling cascade. However, in these studies used pseudotyped HIV-1 virions that bypass the CD4 and CXCR4/CCR5 receptor mediated entry of infectious virions. Therefore, to test the physiological relevance of the link between activation of p38/MSK1 pathway and viral transcription, we carried out infection experiments using infectious HIV-1 virions. We infected THP-1macs with the R5 tropic HIV-1 BAL virions and confirmed productive infection by measuring HIV-1 p24 protein in the cellular lysates by immunoblotting. Results in **Figures 6A,B** showed that cocaine (25 μM) treatment increased the levels of p24 protein relative to untreated cells. These results support the potentiating effects of cocaine on viral transcription that was observed with pseudotyped HIV-1 virions (**Figure 5**). Then, we measured the activation of p38 MAPK/MSK1/H3 ser10 signaling cascade in the cellular lysates of THP1macs infected with HIV-1 BAL virions (**Figures 6C,D**). These data revealed that cocaine treatment induced phosphorylation of p38 MAPK, MSK1, and H3 Ser10 and provide further evidence that cocaine treatment activates p38 MAPK/MSK1/H3 ser10 signaling cascade in macrophages.

### Cocaine-Induced Activations of p38-MAPK/MSK1 Pathway Leads to Enhanced HIV-1 Transcription in Primary MDMs

**FIGURE 7:**
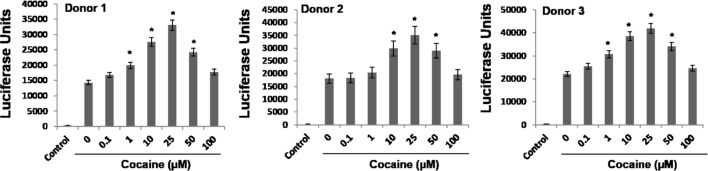
**Cocaine enhances HIV-1 transcription in primary MDMs.** Primary monocytes were isolated from blood of three different donors by Ficoll-based separation method and differentiated into MDMs using M-CSF. MDMs were inoculated with pseudotyped HIV-1 virions and then cultured in the absence or presence of 1–100 μM cocaine. Viral replication in MDMs was monitored 48–72 hpi by measuring the luciferase activity in the cell lysates. The results are expressed as mean ± SE for three independent experiments for each donor. ^∗^*p* < 0.05 is for the comparison of cocaine treated cells vs. untreated cells.

Differentiated THP-1 monocytic cells (THP-1macs) used in this study is an excellent model system and has been extensively used to study signaling pathways of macrophages (Nong et al., 1991; Gomez-Marin et al., 1998; Dreskin et al., 2001; Vuletic et al., 2011). This is mainly because the differentiated THP1macs adhere, stop proliferating, demonstrate increased phagocytosis, and other macrophage physiology (Nong et al., 1991; Gomez-Marin et al., 1998; Dreskin et al., 2001; Vuletic et al., 2011). These attributes of THP-1macs have also been useful in understanding HIV-1 biology in macrophages (Nong et al., 1991). However, being a human monocytic leukemia cell line, results using THP-1macs may not exactly recapitulate the signaling cascades of primary macrophages. Therefore, we tested the effects of cocaine on p38 MAPK, MSK1, and H3 Ser10 in primary monocyte-derived macrophages (MDMs). Primary monocytes were isolated from PBMCs of three healthy donors and were differentiated into macrophages by treatment with M-CSF (10 ng/mL) for 5–7 days. These MDMs were inoculated with VSV-G-pseudotyped HIV-1-Luc reporter virus and then cultured in the absence or presence of cocaine (1–100 μM). HIV-1 LTR promoter drive transcription in the MDMs were determined by assaying the luciferase activity in the cell lysates 48–72 hpi. Results from these assays reveal that cocaine treatment increased the HIV-1 LTR-driven transcriptional activity in the MDMs in a dose dependent manner, with 25 μM concentration inducing the highest enhancement (**Figure 7**). These results are comparable to the HIV-1 transcription levels determined in cocaine-treated THP-1macs (**Figure 1**). Collectively, these data further emphasize that cocaine enhances HIV-1 transcription in macrophages.

**FIGURE 8:**
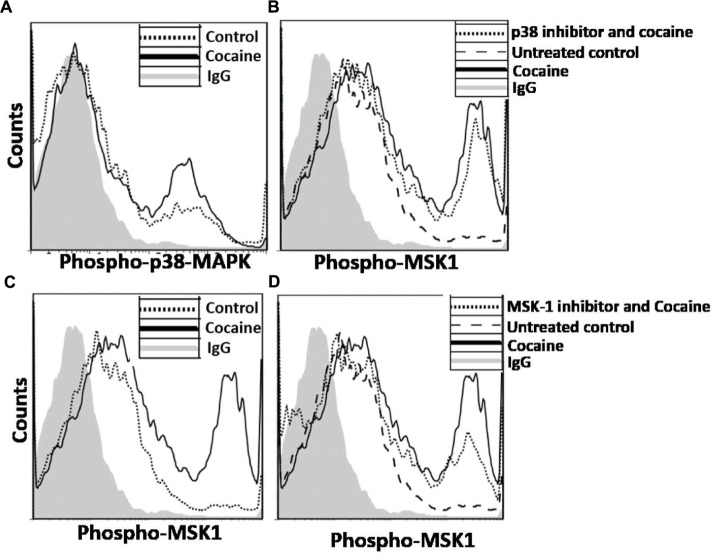
**Induction of p38 MAPK/MSK1 signaling cascade in MDMs.** MDMs were cultured in the absence or presence of 25 μM cocaine and analyzed by flow cytometry to determine the levels of **(A)** phosphorylated p38 MAPK and **(B)** phosphorylated MSK1. To determine that cocaine specifically targets p38 MAPK/MSK1 pathway, MDMs were pretreated with 10 μM p38 MAPK inhibitor (SB-203580) or 10 μM MSK1 inhibitor (SB-747651A) prior to cocaine treatment. Thereafter, cells were harvested and analyzed by flow cytometry to determine the **(C)** effects of p38 MAPK inhibitor on MSK1 phosphorylation, and **(D)** effects of MSK1 inhibitor on MSK1 phosphorylation.

We then sought to determine whether the cocaine-induced enhancement in HIV-1 transcription in MDMs is mediated via the activation of the p38 MAPK/MSK1 signaling cascade in primary macrophages as has been the case in the THP-1macs. To test this, we treated MDMs with cocaine and measured phosphorylation of p38 MAPK and MSK1 by flow cytometry. Data from these analyses show that MDMs treated with 25 μM cocaine showed significantly elevated levels of phospho-p38 (**Figure 8A**) and phospho-MSK1 levels (**Figure 8B**), relative to the untreated cells. In line with the experiments with THP-1macs, the specificity of the activation of p38 MAPK/MSK1 pathway in cocaine-treated MDMs was investigated by using pharmacological inhibitors targeting p38 MAPK (SB-203580) or MSK1 (SB-747651A). Pretreatment with 10 μM concentration of the p38 MAPK inhibitor (**Figure 8C**) or the MSK1 inhibitor (**Figure 8D**) abrogated the accumulation of phospho-MSK1 in cocaine-treated MDMs. These findings are in agreement with the data obtained from experiments using THP-1macs (**Figure 3**) and thus strongly support our hypothesis that cocaine activates p38 MAPK/MSK1 signaling in macrophages.

## Discussion

There are approximately ∼36 million individuals currently infected with HIV-1 worldwide^1^ (CDC, 2012). Substance abuse serves as a powerful cofactor at every stage of the ongoing global HIV-1 pandemic (Friedman et al., 2006; Sohler et al., 2007; Cofrancesco et al., 2008; Kipp et al., 2011). Clinical studies suggest that substance abuse is associated with higher risk of infection, increased viral load, accelerated disease progression and worsening of AIDS-related mortality (Anthony et al., 1991; Arnsten et al., 2001, 2002; Friedman et al., 2006; Sohler et al., 2007; Cofrancesco et al., 2008; Kipp et al., 2011). Cocaine is a commonly abused drug among HIV-1 infected patients (Pence et al., 2008) and studies suggest an association between cocaine use and worsening of HIV disease (Chaisson et al., 1989; Anthony et al., 1991; Siddiqui et al., 1993; Larrat et al., 1996; Cofrancesco et al., 2008; Cook et al., 2008; Baum et al., 2009; Cook, 2011). The worsening effects of cocaine abuse is much pronounced on HIV-1 neuro-pathogenesis given that both cocaine and the virus target the CNS (Buch et al., 2012).

HIV-1 infection in the brain results in a range of neurological dysfunctions that are broadly termed as HAND (McArthur et al., 2010). Neuronal apoptosis and neuro-inflammation in the infected brain causes CNS dysfunction and injury (Kaul et al., 2001). Even though the exact mechanism remains largely unclear, since many of the drugs used in the ART regimen do not penetrate the blood brain barrier (Ene et al., 2011), persistent viral replication in the brain has been suggested to play a key role for HAND pathogenesis (Ene et al., 2011). Although neurons are refractory to HIV-1 infection (van de Bovenkamp et al., 2002), microglia and resident macrophages support HIV-1 infection in the brain (Koppensteiner et al., 2012). It has been proposed that cocaine’s potentiating effects on HIV-1 replication in macrophages (Dhillon et al., 2007), play critical roles in worsening of HIV-associated neurological complications in drug abusing patients (Gonzalez-Scarano and Martin-Garcia, 2005). However, the underlying cellular signaling pathways that contribute to HIV-1 infection and replication in the brain, specifically in the macrophages, are not clearly delineated.

Cocaine is known to target several signaling pathways including MAPK, PI3K/Akt, and MSK1, which can modulate transcription of cellular genes (Yao et al., 2009; Yang et al., 2010; Mannangatti et al., 2015). Importantly, MAPK and MSK1 signaling has also been shown to modulate HIV-1 replication, by stimulating transcription from the viral LTR promoter (Muthumani et al., 2004; Horie et al., 2007; Herbein et al., 2010). For instance, HIV-1 transcription is induced by an activated p38 MAPK in macrophages (Kadoki et al., 2010), while inhibition of p38 MAPK in PBMCs and chronically infected monocytic cell lines diminished HIV-1 production (Shapiro et al., 1998). Further in mammalian cells, p38 MAPK is phosphorylated in response to a variety of environmental and cellular insults including pathogens, heat shock, growth factors, osmotic shock, ultraviolet irradiation, and cytokines (Cano and Mahadevan, 1995). Accordingly, p38 MAPK is a key modulator of HIV gene expression in response to UV radiation (Taher et al., 1999). Since cocaine is also known to modulate the p38 MAPK pathway (Yao et al., 2009; Yang et al., 2010), we hypothesized that p38 MAPK is involved in cocaine-induced activation of HIV-1 transcription in macrophages. Our results show that cocaine treatment increased HIV-1 transcription both in in THP1macs (**Figure 1**) and primary MDMs (**Figure 7**). The concentrations of cocaine used in this study ranged from 0.1 to 100 μM. These concentrations were selected to mimic the varied range of plasma concentrations of cocaine reported among drug users (Van Dyke et al., 1976; Mittleman and Wetli, 1984; Peretti et al., 1990; Karch et al., 1998; Blaho et al., 2000; Heard et al., 2008). The phosphorylation status of p38 MAPK was also elevated in cocaine treatment (**Figures 2** and **8**), thus indicating that cocaine augments p38 MAPK activity in macrophages. The activation of p38 MAPK paralleled the increase in HIV-1 transcription. Accordingly, the cocaine-induced enhancement of HIV-1 transcription was abrogated when infected cells were pretreated, prior to cocaine exposure, with p38 MAPK inhibitor (**Figure 5**). These results strongly suggested that cocaine’s potentiating effects on HIV-1 transcription is dependent on activation of p38 MAPK in macrophages.

Once p38 MAPK is activated by phosphorylation, it can target and activate a range of downstream effector proteins including several transcription factors and kinases that are involved in the control of gene expression (Tiedje et al., 2014; Yang et al., 2014). One such target of p38 MAPK is MSK1 (McCoy et al., 2005; Vermeulen et al., 2009), which can directly activate transcription factors such as NF-κB- a well-known stimulator of HIV-1 transcription (Nabel and Baltimore, 1987). Furthermore, strong activation of MSK1 has also been reported in response to cocaine, resulting in gene regulation critical for long-term neuronal alterations leading to drug addiction (Brami-Cherrier et al., 2005). Therefore, we investigated whether cocaine-induced activation of p38 MAPK altered the status of MSK1 activity in macrophages. MSK1 phosphorylation was enhanced in cocaine-treated THP-1macs (**Figures 2D–F**) and MDMs (**Figure 8B**), which juxtaposed well with the activation of p38 MAPK. Pharmacological inhibition of p38 MAPK prevented phosphorylation of MSK1 in cocaine-treated THP-1macs (**Figure 3**) and MDMs (**Figure 8**). These data reveal that MSK1 is a critical mediator of the p38 MAPK intracellular signaling cascade in response to cocaine in macrophages.

The transcription of eukaryotic genes is regulated by remodeling of chromatin structure (Fischle et al., 2003). Histone H3 is a major nucleosomal protein involved in the chromatin organization in the eukaryotic cells (Fischle et al., 2003). To initiate transcription, the N-terminal tail of histone H3 undergoes several modifications that induce changes in the chromatin environment (Fischle et al., 2003). The phosphorylation on serine 10 and acetylation on lysine 14 of histone H3 have been commonly associated with genes that are being actively transcribed (Fischle et al., 2003). MSK1, a key signaling molecule downstream of p38 MAPK (McCoy et al., 2005; Vermeulen et al., 2009), is known to induce phosphorylation of H3 Ser10, thereby resulting in transcriptional activation of cellular genes (Soloaga et al., 2003). A recent study also suggested that H3 Ser10 phosphorylation drives HIV-1 transcription (Sahu et al., 2015). Therefore, we investigated whether cocaine-mediated activation of p38 MAPK/MSK1 signaling axis targeted H3 Ser10 in macrophages. Cocaine caused a dose-dependent increase in phosphorylation of H3 Ser10 (**Figures 2G–I**), with optimal activation obtained at a concentration of 25 μM cocaine. Fittingly, cells treated with 25 μM cocaine displayed the greatest increase in p38 MAPK/MSK1 signaling and HIV-1 transcription. These data suggest that the positive effect of cocaine on HIV-1 transcription stems most likely from the H3 Ser10 phosphorylation executed by robustly activated p38 MAPK/MSK1 signaling pathway.

**FIGURE 9:**
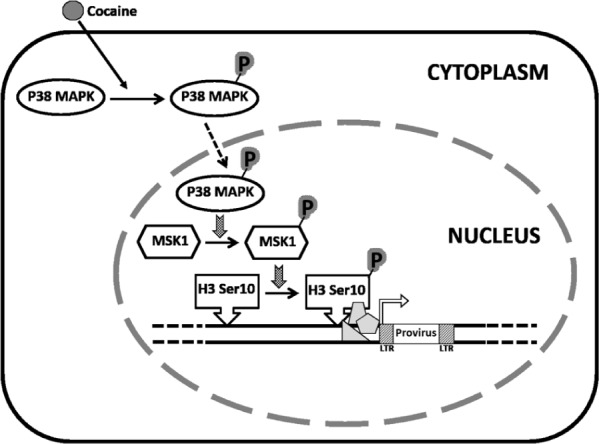
**Model proposing a role for cocaine-induced activation of p38 MAPK/MSK1 signaling pathway in the induction of HIV-1 transcription in macrophages.** We propose that cocaine enhances HIV-1 transcription in macrophages by activating the p38 MAPK/MSK1/H3 Ser10 pathway. Specifically, cocaine activates p38 MAPK in macrophages by inducing phosphorylation of p38 MAPK. Consequently, the activated p38 MAPK induces phosphorylation of the downstream effector MSK1. The activated MSK1 then phosphorylates H3 Ser10, which renders the host chromatin accessible to transcription factors that then stimulate HIV-1 transcription. We hypothesize that cocaine-induced increase in HIV-1 transcription may increase the viral load in the CNS thus contributing to the worsening of HIV-1 neuropathogenesis in drug-abusing HIV-1 infected individuals.

Collectively, our results demonstrate that cocaine enhances HIV-1 transcription by a novel mechanism that involves the p38 MAPK/MSK1/H3 Ser10 pathway (**Figure 9**). We show that cocaine treatment causes activation of p38 MAPK, MSK1, and H3 Ser10 in macrophages. These signaling events likely culminate in the host chromatin, including the integrated provirus, gaining access to transcription factors. The ensuing increase in HIV-1 transcription can promote viral replication in macrophages, accompanied by overproduction of viral neurotoxins in the CNS. However, our results do not address the mechanism by which cocaine activates p38 MAPK. Nevertheless, cocaine is known to bind to the dopamine transporters (DAT; Ritz et al., 1987; Chen et al., 2006), and activate signaling cascades including p38 MAPK (Nair et al., 2005; Lepsch et al., 2009; Yao et al., 2009; Yang et al., 2010). There is some evidence that macrophages respond to dopamine signaling (Gaskill et al., 2013, 2014). Therefore, it is plausible that cocaine activates p38 MAPK in macrophages by utilizing the dopaminergic signaling pathway. However, in the absence of data describing expression of DAT on human macrophages, whether binding of cocaine to DAT in these cells can activate p38 MAPK is yet to be tested. Alternatively, based on the lipophilic properties, cocaine can cross the plasma membrane in these cells and induce p38 MAPK activation via mechanisms that are yet to be clearly define. Currently, studies are underway to test these hypotheses.

## Conclusion

The results presented in this report provide new insights into the complex interaction between drug abuse and HIV-1 replication and describe a potential mechanism by which cocaine exacerbates HIV-1 neuropathogenesis. Specifically, our results highlight that cocaine enhances HIV-1 transcription in macrophages by targeting p38 MAPK/MSK1/H3 Ser10 pathway. Given that macrophages support HIV-1 replication in the CNS, these results suggest that cocaine abuse potentially contributes to the persistent HIV-1 replication and increased viral load in the brain of infected patients. It is important to point out that persistent HIV-1 replication albeit at low levels plays a critical role in HAND pathogenesis that affects a large percentage of HIV-1 infected patients (Vivithanaporn et al., 2010). Therefore, our data may have implications in reducing the burden of HAND and may pave the way in identifying novel cellular pathways that could be targeted for the treatment of HAND pathogenesis in drug abusing patients.

## Author Contributions

CS, AR, and JP carried out the all the experiments. CD and JP designed and directed the entire study. JP, MB, and CD analyzed the data. JP and CD wrote the manuscript and all the authors reviewed the manuscript.

## Conflict of Interest Statement

The authors declare that the research was conducted in the absence of any commercial or financial relationships that could be construed as a potential conflict of interest.

## Footnotes

1 http://www.who.int/mediacentre/factsheets/fs360/en/index.html
